# Rubella and Congenital Rubella Syndrome Control and Elimination — Global Progress, 2000–2012

**Published:** 2013-12-06

**Authors:** Alya J. Dabbagh, Laure Dumolard, Marta Gacic-Dobo, Gavin B. Grant, Susan E. Reef

**Affiliations:** Dept of Immunization, Vaccines, and Biologicals, World Health Organization, Geneva, Switzerland; Global Immunization Div, Center for Global Health, CDC

Rubella virus usually causes a mild fever and rash in children and adults.* However, infection during pregnancy, especially during the first trimester, can result in miscarriage, stillbirth, or infants with congenital malformations, known as congenital rubella syndrome (CRS). In 2011, the World Health Organization (WHO) updated guidance on the preferred strategy for introduction of rubella-containing vaccine (RCV) into national routine immunization schedules with an initial wide-age-range vaccination campaign that includes children aged 9 months–15 years (1). WHO also urged all member states to take the opportunity offered by accelerated measles control and elimination activities as a platform to introduce RCVs (1). The Global Measles and Rubella Strategic Plan (2012–2020) published by the Measles Rubella Initiative partners in 2012 and the Global Vaccine Action Plan endorsed by the World Health Assembly in 2012 include milestones to eliminate rubella and CRS in two WHO regions by 2015, and eliminate rubella in five WHO regions by 2020. This report summarizes the global progress of rubella and CRS control and elimination during 2000–2012. As of December 2012, a total of 132 (68%) WHO member states had introduced RCV, a 33% increase from 99 member states in 2000. A total of 94,030 rubella cases were reported to WHO in 2012 from 174 member states, an 86% decrease from the 670,894 cases reported in 2000 from 102 member states. The WHO Region of the Americas (AMR) and European Region (EUR) have established rubella elimination goals of 2010 and 2015, respectively. AMR has started to document the elimination of measles, rubella, and CRS; in EUR, rubella incidence has decreased significantly, although outbreaks continue to occur.

## Immunization Activities

Data were obtained from the WHO and United Nations Children’s Fund (UNICEF) Joint Reporting Form (JRF), which is used to collect information from United Nations member states on vaccination campaigns, vaccination schedules, and number of doses of RCV administered by routine immunization services (2). Data from 2000–2012 were analyzed to assess the changes in rubella and CRS control activities.

**FIGURE 1:**
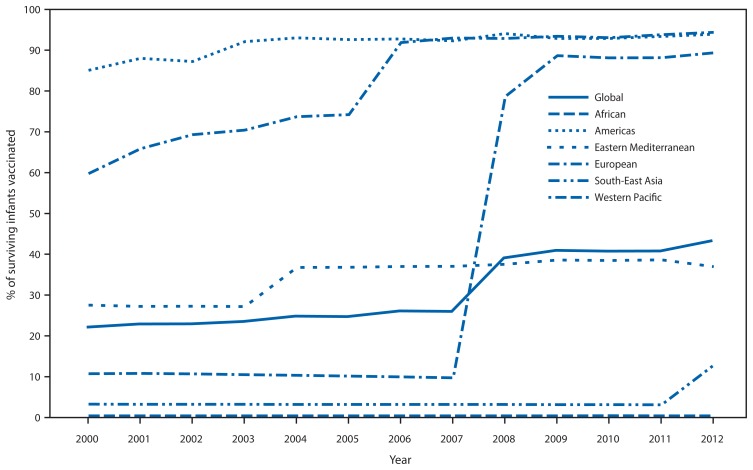
Proportion of surviving infants receiving rubella-containing vaccine (RCV) — World Health Organization (WHO) regions, 2000–2012^*^ ^*^ Based on WHO–United Nations Children’s Fund (UNICEF) estimates of rubella coverage. Note: China introduced RCV into its immunization schedule in 2008.

As of December 2012, a total of 132 (68%) of the 194 member states had introduced RCV: three (7%) in the African Region (AFR), 35 (100%) in AMR, 14 (64%) in the Eastern Mediterranean Region (EMR), 53 (100%) in EUR, five (45%) in the South-East Asia Region (SEAR), and 22 (81%) in the Western Pacific Region (WPR). Member states with RCV in their schedule accounted for 59% of the global population in 2012, up from 31% in 2000. The proportion of infants who received a RCV dose was 22%† in 2000 to 43% in 2012, a 96% increase (Figure 1).

**FIGURE 2:**
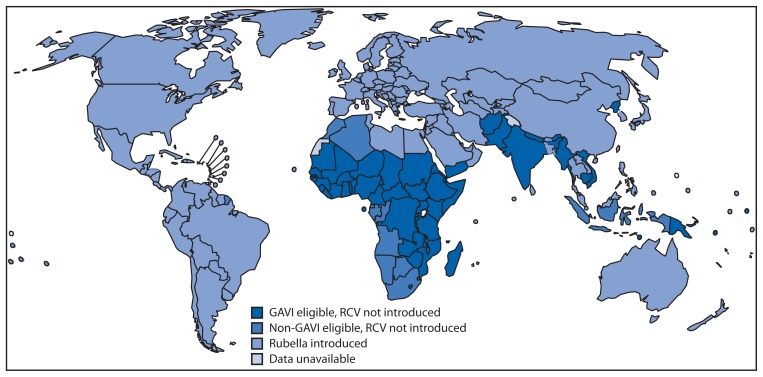
World Health Organization member states that have introduced rubella-containing vaccine (RCV) and member states potential to introduce RCV with GAVI Alliance support,^*^ 2012 ^*^ Additional information about the GAVI Alliance, formerly the Global Alliance for Vaccines and Immunisation, and the support it provides, is available at http://www.gavialliance.org.

During 2000–2012, of the 33 member states introducing RCV, one is in AFR, four in AMR, two in EMR, 13 in EUR, three in SEAR, and 10 in WPR. A wide-age-range campaign was part of the implementation for introduction in 23 member states. One member state in the past 10 years interrupted RCV use and plans to reintroduce RCV. Of the 62 member states that had not introduced RCV into their national immunization program by the end of 2012, 50 (81%) are eligible for GAVI Alliance support (Figure 2).§ Eligibility requirements include measles coverage >80% and a gross national income per capita ≤1,550 U.S. dollars.

Of 132 member states that have introduced RCV, 124 (94%) provide the first RCV dose with the first routine dose of measles-containing vaccine (MCV) and eight (6%) provide the first RCV dose with the second MCV dose. In 2012, the first RCV dose was administered at age 9 months in eight (6%) member states, age 12–18 months in 120 (91%) member states, and age >18 months in three (3%) member states. RCV is provided in combination with measles vaccine alone in 11% of member states and in combination with measles and mumps (with or without varicella vaccine) in 89% of member states.

## Surveillance Activities

Rubella and CRS surveillance are necessary to evaluate the disease burden before and after introduction of RCV, and to identify pregnant women infected with rubella and children with CRS who require follow-up. The JRF collects surveillance data from member states, including cases of rubella and congenital rubella syndrome; for this report, data from 2000–2012 were analyzed. WHO has published case definitions for rubella and CRS as recommended standards for member state reporting (3). The number of member states reporting rubella cases increased from 102 in 2000 to 174 in 2012. The number of member states reporting CRS cases increased from 75 in 2000 to 129 in 2012. Of 132 member states that introduced RCV before 2012, 129 (98%) had reported rubella cases and 121 (92%) had reported CRS surveillance results in the previous 5 years. Of the 62 member states that had not introduced RCV before 2012, 60 (97%) had reported rubella cases and 49 (79%) had reported CRS cases in the previous 5 years (Table). In 2012, substantially more cases were reported in EUR (30,536 cases) and WPR (44,275 cases) than in other regions (19,219 cases). Rubella outbreaks with >2,000 cases were reported during 2012 in Romania (4), Japan (5), and Poland (6). These outbreaks occurred in member states with established rubella control programs, and where RCV introduction focused initially on vaccination of females.

Rubella elimination targets have been established in AMR and EUR. In AMR, the last endemic rubella and CRS case was reported in 2009, and the region is documenting the elimination of rubella and CRS. In EUR, the number of rubella cases decreased by 95%, from 621,039 in 2000 to 30,536 in 2012; however, cases increased from 9,672 in 2011.

### Editorial Note

Following a period of steady but slow increases in rubella control, a new phase of accelerated rubella control and CRS prevention has begun, marked by the 2011 WHO position paper recommending a strategy to eliminate rubella and CRS, and emphasizing RCV introduction in all member states and the linkage of rubella to measles control activities. Programmatic integration of RCV into an existing measles schedule is straightforward, involving no increase in the number of injections or in cold-chain requirements with a combined measles-rubella vaccine, no change in age of vaccine administration, and minimal change in recording and reporting formats. Sustainable financing from government and partners is required to introduce and maintain routine rubella immunization activities and inclusion of RCV for all measles campaigns following introduction. The additional cost to include the rubella antigen with the measles vaccine is 0.199 to 0.309 U.S. dollars per dose.¶ GAVI Alliance funding is available for eligible member states to support introduction; the funding supports a grant for introduction of RCV into the national routine immunization schedule and a wide-age-range RCV campaign. Nine member states applied for these funds in 2012.

For RCV introduction to succeed, decision makers need to identify rubella and CRS as a public health priority, provide sustainable support, and ensure adequate coverage. Suboptimal implementation of rubella control strategies might result in an increase in CRS cases; following years of low vaccine coverage and lower levels of rubella virus transmission, persons who would have been infected as children remain susceptible until they reach adolescence and adulthood, resulting in a potential increase in CRS cases, as seen in Greece (7). To prevent an increase in rubella and CRS, the preferred RCV introduction strategy is to first conduct a national wide-age-range campaign and then immediately introduce RCV into the routine immunization schedule. Postcampaign coverage surveys validate the campaign coverage and can identify potential gaps.

Activities to reach elimination goals in AMR and EUR have decreased the number of cases in 2012 relative to 2000. Improvements in surveillance have not been consistent between member states and WHO regions. Improved surveillance for rubella in AFR and SEAR has increased the number of rubella cases detected that previously would have been undetected. Strong reporting systems in WPR and EUR resulted in a greater proportion of the cases reported globally. In AMR, a clear decrease in rubella cases is associated with a decrease in CRS cases.

Outbreaks in EUR and WPR indicate that while control and elimination activities are ongoing, some member states within these regions are at risk for large outbreaks. Initiation of CRS control activities focused on vaccinating girls and women, which decreased rubella virus transmission but resulted in a large proportion of susceptible persons, especially males. A large population susceptible to rubella infection (primarily males) has a high risk for outbreak and transmission of rubella virus to unvaccinated pregnant women. Surveillance for rubella infection benefits from integration with measles surveillance systems, but additional effort is required to strengthen the system to ensure that febrile rash illness cases reported in pregnant women or their immediate contacts are fully investigated, including ascertaining pregnancy outcome. Surveillance to detect CRS is needed to monitor the impact of vaccination.

What is already known on this topic?

Rubella virus infection during pregnancy, especially during the first trimester, can cause miscarriage, stillbirth, or congenital rubella syndrome (CRS). The World Health Organization (WHO) recommends that all member states introduce rubella-containing vaccines (RCVs) to control rubella and CRS. The World Health Assembly has set two goals: rubella elimination in two WHO regions by 2015 and measles and rubella elimination in five WHO regions by 2020.

What is added by this report?

The number of countries using RCVs in their immunization program and reporting rubella and CRS surveillance data has steadily increased from 2000 to 2012. As of December 2012, a total of 132 (68%) WHO member states had introduced RCV, a 33% increase from 99 member states in 2000. A total of 94,030 rubella cases were reported to WHO in 2012 from 174 member states, an 86% decrease from the 670,894 cases reported in 2000 from 102 member states.

What are the implications for public health practice?

Near elimination of rubella and CRS in the Americas proves that the tools exist to make elimination possible, and substantial progress is being made globally. However, gaps in surveillance limit the ability to monitor progress toward elimination, and recent outbreaks in Europe and Asia demonstrate the need for sustained, high-quality immunization programs.

The difference between the 2012 global coverage with the first dose of MCV (83%) and RCV (43%) highlights the extent of the opportunity missed by the lack of integration of RCV with MCV. With a new phase of rubella control, member states should consider introducing or strengthening RCVs immunization activities and strengthening their existing rubella and CRS surveillance systems. The availability of technical expertise and financial resources from Measles Rubella Initiative partners, including the GAVI Alliance, provides a foundation to accelerate rubella control and CRS prevention activities globally. In addition, political commitment at the federal, provincial, and district levels is needed to reach the Measles Rubella Initiative and Global Vaccine Action Plan goal of elimination in five WHO regions by 2020.

## Figures and Tables

**TABLE:** Global progress in rubella and congenital rubella syndrome (CRS) control and elimination activities — World Health Organization (WHO) regions, 2000 and 2012

WHO region	2000						2012						
	
	Member states with rubella-containing vaccine in schedule		Member states reporting		No. of reported cases		Member states with rubella-containing vaccine in schedule		Member states reporting		No. of reported cases		Control target*
					
	No.	(%)	Rubella	CRS	Rubella	CRS	No.	(%)	Rubella	CRS	Rubella	CRS	
Africa (46 member states)	2	(4)	7	3	865	0	3	(6)	41	20	10,830	69	None
Americas (35 member states)	31	(89)	25	18	39,228	80	35	(100)	35	35	21	3	Elimination
Eastern Mediterranean (22 member states)	12	(55)	11	6	3122	0	14	(64)	18	9	1,698	20	None
Europe (53 member states)	40	(75)	41	34	621,039	48	53	(100)	46	42	30,536	60	Elimination
South-East Asia (11 member states)	2	(18)	3	2	1,165	26	5	(45)	11	6	6,670	14	None
Western Pacific (27 member states)	12	(44)	15	12	5,475	3	22	(81)	23	17	44,275	134	Control
**Global (194 member states)**	**99**	**(51)**	**102**	**75**	**670,894**	**157**	**132**	**(68)**	**174**	**129**	**94,030**	**300**	**None**

**Source:** WHO–United Nations Children’s Fund (UNICEF) Joint Reporting Form.

* No control targets were set before 2000.

## Footnotes

* Additional information available at http://www.cdc.gov/vaccines/vpd-vac/rubella/in-short-adult.htm.

† Based on 2012 UNICEF–WHO joint estimate adjusted for 2012 United Nations Development Programme calculations of surviving infants per region, available at http://www.who.int/immunization_monitoring/data/en.

§ Additional information about the GAVI Alliance, formerly the Global Alliance for Vaccines and Immunisation, and the support it provides, is available at http://www.gavialliance.org.

¶ UNICEF vaccine price information for measles and measles-rubella vaccine available at http://www.unicef.org/supply/index_57476.html.
